# Portal Hypertension in Childhood Bilateral Wilms' Tumor Survivor: An Excellent Indication for TIPS

**DOI:** 10.1155/2013/523154

**Published:** 2013-04-04

**Authors:** Ghassan Nakib, Valeria Calcaterra, Marco Brunero, Ilaria Goruppi, Pietro Quaretti, Lorenzo Paolo Moramarco, Raffaele Bruno, Alessandro Raffaele, Gloria Pelizzo

**Affiliations:** ^1^Department of the Mother and Child Health, Pediatric Surgery Unit, IRCCS Policlinico San Matteo Foundation and University of Pavia, Piazzale Golgi 2, 27100 Pavia, Italy; ^2^Department of the Mother and Child Health, Pediatric Unit, IRCCS Policlinico San Matteo Foundation and Department of Internal Medicine University of Pavia, 27100 Pavia, Italy; ^3^Unit of Interventional Radiology, IRCCS Policlinico San Matteo Foundation, 27100 Pavia, Italy; ^4^Infectious Diseases Department, IRCCS Policlinico San Matteo Foundation and University of Pavia, 27100 Pavia, Italy

## Abstract

*Introduction*. Increased pressure in portal venous system is relatively a rare complication after chemoradiotherapy for Wilms' tumor (WT). In paediatric population, feasibility and efficacy of transjugular intrahepatic portosystemic shunt (TIPS) in portal hypertension nonresponsive to medical or endoscopic treatment have been recently advocated. We report a case of TIPS positioning in a 15-year-old girl with portal hypertension as a long-term sequel of multimodality therapy in bilateral WT. *Case Report*. Two-year-old girl was diagnosed for bilateral WT. Right nephrectomy with left heminephrectomy and chemoradiotherapy were performed. At 7 years of age, the first gastrointestinal bleeding appeared, followed by another episode two years later, both were treated successfully with beta-blockers. At 15 years of age, severe unresponsive life-threatening gastroesophageal bleeding without hepatosplenomegaly was managed by TIPS. Reduction of the portosystemic pressure gradient was obtained. *Conclusion*. TIPS positioning for portal hypertension in long-term tumors' sequel is feasible and could be considered as an additional indication in paediatric patients.

## 1. Introduction

Perinatal events like umbilical catheterization, thrombophilic states, tumors, and abdominal infection are the main causes of hepatic injury leading to portal hypertension (PH) [1]. Children submitted to chemoradiotherapy for Wilms' tumor (WT) could also end in PH caused by portal vein fibrosis [2].

A wide range of therapeutic modalities, including medical and surgical treatments for PH, has been adopted. The transjugular intrahepatic portosystemic shunt (TIPS) has been introduced as an alternative safe management for complicated PH even in pediatrics [3, 4].

We report a unique case of TIPS positioning for PH observed in a 15-year-old girl subsequent to multimodality therapy for bilateral WT. 

## 2. Case Report

A 2-year-old girl underwent right nephrectomy and left heminephrectomy for bilateral WT. Chemotherapy (Vincristine and Actinomycin D) and radiotherapy were done.

At 9 years of age, she developed the first episode of melena. Gastroscopy showed oesophageal white F1 varices. No hepatosplenomegaly or dilatation of portal vein was documented on CT scan. Two years later melena and hematemesis reappeared. Gastroscopy evidenced oesophago-gastric varices (F2 blu, red wale markings). Liver biopsy showed periportal fibrosis and portal interlobular venules obliterations, without signs of cirrhosis. Beta-blockers treatment was adopted with resolution of the gastrointestinal bleeding, and oesophageal varices banding were not necessary. 

Between 11 and 15 years of age, the patient was on followup without any recurrences of the bleeding. 

At 15 years of age, a new severe bleeding episode occurred. On endoscopy, oesophageal varicose veins had transsphinteric involvement, and elastic ligature failed. Medical therapy was successful.

**Figure 1:**
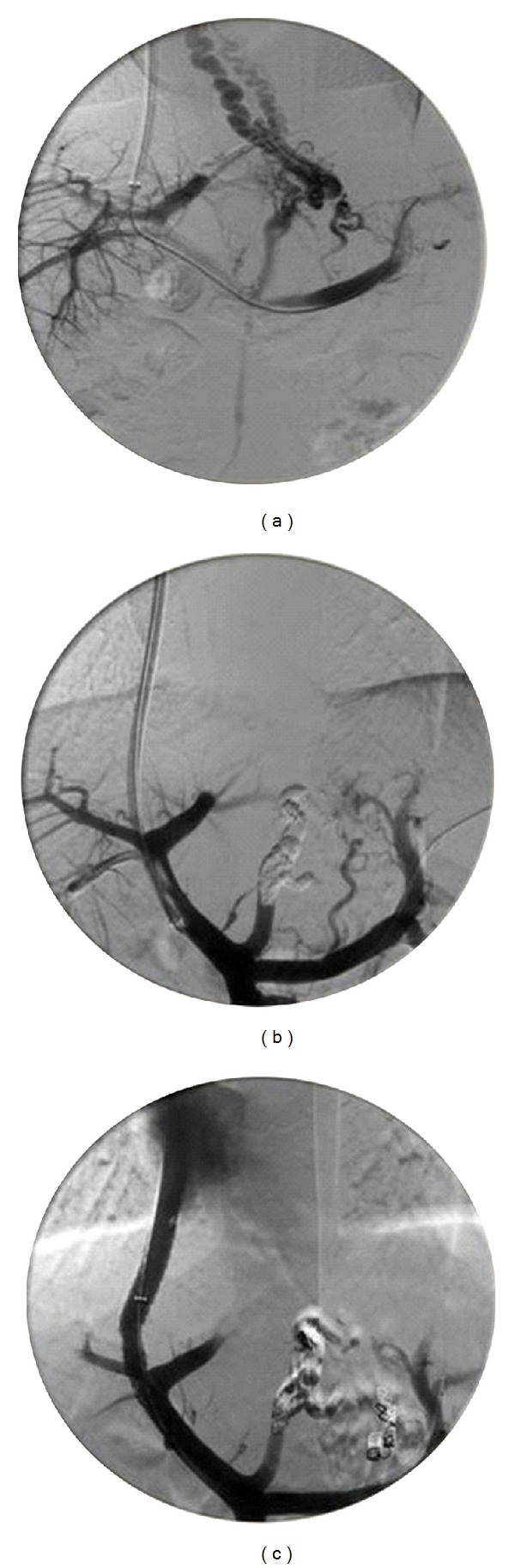
Portohepatic venous system angiography: (a) contrast imaging of the gastrooesophageal varices; (b) sclerosis of the varices; and (c) evaluation of and transintrahepatic portosystemic shunt positioning.

Magnetic resonance angiography showed no variation in the splenoportal vascular system. No changes found on liver biopsy. A rebleeding (Hb 5 gr/dL) occurred few days later, and endoscopic varices sclerosis was performed. After stabilization of clinical conditions, portosystemic shunting was done by means of TIPS. The right hepatic vein was catheterized via right jugular access. The hepatic venous portal gradient measured by means of a balloon catheter (OB catheter 8.5 mm, Boston, MA, USA) was inflated and deflated three times and was within normal range (<5 mmHg) confirming the prehepatic origin of PH. The right branch of intrahepatic portal vein was reached, and the true portoatrial gradient pressure of 14 mmHg was registered. For the life-threatening hemorrhage from gastric varices, embolization of the giant left gastric vein with coils and Glubran mixed to Lipiodol was carried on before reversing the hepatofugal flow of the varices (Figures 1(a) and 1(b)). The shunt was completed by placing a PTFE Viatorr stent (8 mm × 60 mm) to maintain the tract between the portal and the hepatic veins (Figure 1(c)). 

**Figure 2:**
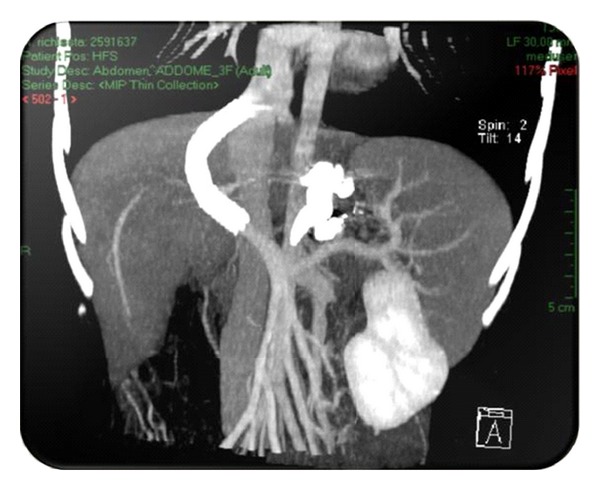
Angiographic TC after transintrahepatic portosystemic shunt positioning.

On endoscopy, oesophago-gastric varices disappeared six months after TIPS positioning. Laboratory investigations were normal. On Doppler ultrasound and angiographic CT (Figure 2), patency of the inserted shunt with normal peak flow velocity was registered.

## 3. Discussion

WT is the commonest kidney cancer in children. It represents 55% of all abdominal tumors and 6% of all cancers under 15 years of age. Bilateral WT involves approximately 5–7% of patients. Treatment includes surgery and chemoradiotherapy. The cure rate for WT is >85% of overall survival rate, but late side-effects after therapy are reported [2, 5]. Liver damage with PH may occur in subjects who received either radiation therapy or actinomycin D or both [2].

The pressure in portal venous system could be raised either due to an obstruction in the extrahepatic portal venous system or due to increased resistance to portal blood flow. This resistance can occur commonly at the level of sinusoid or proximal to it. In the literature, this complication has been reported only in few cases of bilateral WT. Venoocclusive syndrome at the postsinusoidal level has been implicated as a cause of PH in WT patients [2].

Most evidence of portal hypertension is the development of oesophageal and gastric varices. The probability of bleeding in grade II or III arises up to 85%. Vasoactive drugs or endoscopic treatment are effective in the control of bleeding up to 90% of cases. Surgery and TIPS should be reserved for those unresponsive to the combined therapy [1, 6, 7]. 

Establishing a side-to-side portacaval shunt by TIPS, portosystemic pressure gradient in over 90% of cases is reduced, and excellent haemostatic effect (95%), with low rebleeding rates (<20%), could be obtained [6].

Indications for urgent TIPS positioning are persistent bleeding and refractory ascites unresponsive to combined endoscopic and pharmacological therapies. TIPS in children is recommended when: (1) surgical shunting is not feasible; (2) bad prognosis is predictable; (3) medical complications jeopardise surgical shunting outcome; and (4) end-stage liver failure requires a bridge to transplantation [3, 7].

Technical success rates of TIPS in children ranges from 78% to 100% and is comparable to the adult literature [4]. Pathologies that could benefit from tips are reported in Table 1. In childhood cancer survivors, signs of hepatic injury are encountered at a median followup of 12 years. The reported prevalence of hepatic late adverse effects varied between 8 and 52.8%. This wide range is due to variable distribution of risk factors for liver damage and to different regimens of the treatment [8]. 

Our patient had recurrent severe oesophageal-gastric bleeding, unresponsive to endoscopic and medical treatments. No signs of hepatosplenomegaly were present. Prehepatic nature of PH consistent with portal vein fibrosis was suspected; the measurement of hepatic venous portal gradient confirmed this origin. TIPS positioning was the only safe and less invasive method. Surgical shunting was at high risk of complications in a patient with a solitary hemileft kidney. Failure meant imminent liver transplant.

No previouse cases for TIPS application in portal hypertension as long-term sequel of bilateral WT have been reported.

Complications rates in children undergoing TIPS are similar to those of adults (4–6). A multidisciplinary approach and long-term followup are essential.

## 4. Conclusion

TIPS in late complicated portal hypertension and in paediatric patients with high-risk surgical shunting seems to be an excellent indication. Our case highlights the importance of PH detection in long-term childhood cancer survivors.

## Figures and Tables

**Table 1:** Pathologies that could benefit from transjugular intrahepatic portosystemic stent shunting.

Prehepatic	
Congenital stenosis of the portal vein	
Extrahepatic portal vein obstruction	
Extrinsic compression of the portal vein	
Intestinal failure associated liver disease in chronic intestinal pseudoobstruction syndrome	
Splenic vein thrombosis	
Portal vein thrombosis	
Portal cavernoma	
Idiopathic portal fibrosis	
Postradiotherapy portal vein fibrosis	

Intrahepatic	

Chronic hepatitis	
Congenital hepatic fibrosis	
Granulomatous diseases	
Hypervitaminosis A	
Nodular regenerative hyperplasia	
Noncirrhotic portal fibrosis	
Venoocclusive disease	
Peliosis hepatitis	
Polycystic disease	
Sclerosing cholangitis	

Posthepatic	

Hepatic vein outflow/inferior vena cava thrombosis (Budd-Chiari syndrome)	
